# Integrated analysis of DNA copy number and gene expression microarray data using gene sets

**DOI:** 10.1186/1471-2105-10-203

**Published:** 2009-06-29

**Authors:** Renée X Menezes, Marten Boetzer, Melle Sieswerda, Gert-Jan B van Ommen, Judith M Boer

**Affiliations:** 1 Center for Human and Clinical Genetics, Leiden University Medical Center, PO Box 9600, 2300 RC Leiden, The Netherlands; 2 Pediatric Oncology Laboratory, Erasmus Medical Center, Rotterdam, The Netherlands; 3 BioRange, Netherlands Bioinformatics Centre, Nijmegen, The Netherlands; 4 Center for Medical Systems Biology, Leiden, The Netherlands

## Abstract

**Background:**

Genes that play an important role in tumorigenesis are expected to show association between DNA copy number and RNA expression. Optimal power to find such associations can only be achieved if analysing copy number and gene expression jointly. Furthermore, some copy number changes extend over larger chromosomal regions affecting the expression levels of multiple resident genes.

**Results:**

We propose to analyse copy number and expression array data using gene sets, rather than individual genes. The proposed model is robust and sensitive. We re-analysed two publicly available datasets as illustration. These two independent breast cancer datasets yielded similar patterns of association between gene dosage and gene expression levels, in spite of different platforms having been used. Our comparisons show a clear advantage to using sets of genes' expressions to detect associations with long-spanning, low-amplitude copy number aberrations. In addition, our model allows for using additional explanatory variables and does not require mapping between copy number and expression probes.

**Conclusion:**

We developed a general and flexible tool for integration of multiple microarray data sets, and showed how the identification of genes whose expression is affected by copy number aberrations provides a powerful approach to prioritize putative targets for functional validation.

## Background

Tumor cells accumulate genetic damage, including changes in DNA copy number, sequence and methylation, resulting in the dysfunctioning of key regulators [1]. The advent of microarray technology has allowed genome-wide monitoring of these molecular changes at the DNA and RNA level. Gene expression profiling has facilitated classification of cancers into biologically and clinically distinct categories [2-7]. High-resolution array-based comparative genomic hybridization (array-CGH) has allowed the delineation of recurrent DNA copy number alterations in tumors [8-10]. Gene dosage changes play an important role in tumor development; oncogenes may be enhanced by DNA amplification and tumor suppressor genes may be inactivated by a physical deletion. Therefore, integrated analysis of both copy number and gene expression microarray data could give additional information about the role of copy number alterations in the development of cancer.

Combined analysis of DNA copy number and gene expression microarrays of the same or similar tumor samples has revealed a major and direct effect of allelic imbalance on gene expression in a variety of cancer types, including breast [11,12], pancreatic [13], colorectal [14], skin [15], head and neck [16,17], prostate [18], multiple myeloma [19], and lung [20] cancer. On a global level, 40–60% of the genes in higher level amplifications showed elevated expression, while circa 10% of highly overexpressed genes were amplified [11,12]. In low-level copy number aberrations, only about 10% of the genes have been reported to show concordant changes in gene expression [11,12,21].

Several approaches have been described to identify those genes whose expression levels are most significantly associated with copy number changes of the corresponding genomic region.

In the context of natural copy number variation in human populations, Stranger and co-authors [22] used a linear regression model to study associations between gene expression and copy number within a 2 Mb window. For the analysis of tumor microarray data, some authors performed a simultaneous exploratory analysis of the different microarray datasets, ordered along the genome, to search for regions where both copy number and gene expression are affected [12,14,23,24], or gene expression and DNA methylation [25]. While this can be clarifying if an effect is found, due to the small effect sizes and the often low signal-to-noise ratio in array data this approach tends to be inefficient. For example, a two-fold change in DNA copy number was observed to be accompanied on average by 1.5-fold changes in mRNA levels in breast tumors [12].

Other cancer studies classified samples according to the presence of chromosomal abnormalities, and subsequently tested for differences in gene expression between altered and unaltered samples. Some studies use a gene-wise test statistic similar to the Student's t-statistic [11,13,16,19] or a one-sided Wilcoxon rank-sums test [25-27]. Garraway and co-authors [15] used supervised analysis looking for gene expression differences between cell lines with and without 3p amplification. Adler and co-authors [28] used a classification approach as the first step in their stepwise linkage analysis of microarray signatures, where they test for differences in copy number between groups of breast cancer samples with and without the wound expression signature. While known and novel tumor-related genes were identified, these approaches may be unable to detect associations between low-level copy number changes and expression variation due to the categorization.

Low-level gains and losses, representing the most common types of genetic alterations in most cancers, were shown to have a significant influence on expression levels of genes in the regions affected, but these effects were more subtle on a gene-by-gene basis [11,21]. However, the impact of low-level gains on the dysregulation of gene expression patterns in cancer may be equally important if not more important than that of high-level amplifications [11-13]. Therefore, the search for DNA regions that might be involved in the initiation and progression of cancer must be powerful enough to detect subtle gene-specific effects that are possibly consistent across many genes. Moreover, the analysis method must take into account the high-dimensionality of the problem, and provide careful control of the error.

We propose to look for associations between copy number and expression not only using individual genes, but also using gene sets. Such a model can improve the power to detect associations, as neighbouring genes may also display association. If different microarray platforms are used to measure copy number and expression, it involves less arbitrariness because no mapping between copy number and expression probes is necessary. To illustrate these points, we first run a simulation study and then apply our model to two publicly-available breast cancer datasets. We will show that the use of gene sets is relevant not only when studying the impact of large-amplitude copy number changes (more than one copy gained or lost), but also in case of more subtle changes, either of low amplitude or spanning a small (<1 Mb) genomic region. The discussion that follows includes other possible applications and useful extensions.

## Results

We wish to find which individual copy number changes affect gene expression levels within the same chromosomal region. For this, we propose to model copy number as a function of the expression levels of many genes at the same time. Statistically significant associations are indicated by a significant p-value for the copy number probe, and the genes with expression levels the most associated with this outcome are prioritized in a heatmap. We evaluate the power of this model in particular experimental setups via a simulation study below. After this, we apply both the gene-set model (2) and the gene-to-gene model (1) to experimental datasets.

### Simulation study

**Figure 1:**
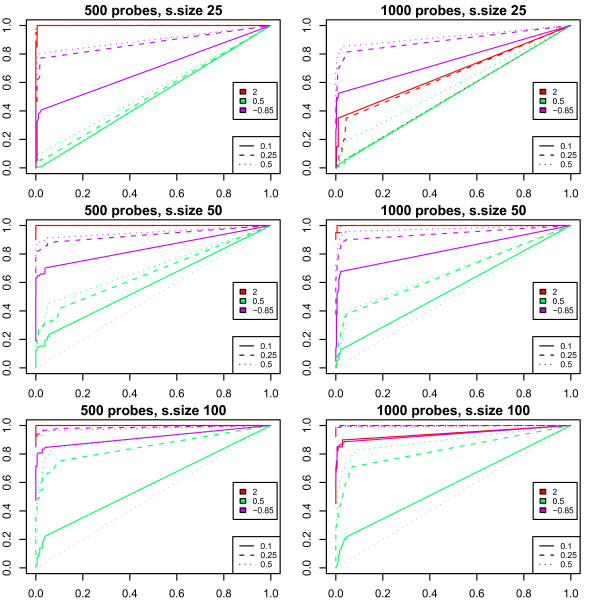
**Receiver-operator characteristic curves for the simulation study**. Conditions shown are: sample size 25, 50 and 100 (rows from top to bottom); length of the studied region 500 and 1000 probes (columns from left to right); proportion of samples with each effect 10, 25 and 50 percent (solid, dashed and dotted lines); and copy number effect sizes 2, 0.5 and -0.85 (line colours red, green and purple). For details about the study setup, see appendix.

In order to illustrate the power of the model to identify association patterns, we run a simulation study. We assume for simplicity that both copy number and expression measurements are obtained using the same arrays, so that there is a one-to-one correspondence between them. The study is designed to represent various situations commonly encountered in practice, where typically 10–50% of the samples display mild copy number effects spanning a sizable genomic region (here 26% of the total probes), such as part of a chromosome arm, or strong copy number effects spanning a small region (here between 2 and 8% of the total probes), typical of amplifications. Each probe's expression level is assumed to be a function of its own copy number, with various degrees of association. Key parameters were estimated from publicly available datasets, amongst them the amount of variability of copy number and expression measurements, as well as the distribution of the associations between copy number and expression. Sample sizes of 25, 50 and 100 are considered. We evaluate the results by producing receiver-operating characteristic (ROC) curves of the regional model for each case, and consider that an effect is detectable if there is power of at least 60% to detect it using an FDR of 10%. For more details about the study setup, see Appendix. The results are reassuring, as the ROC curves in figure 1 illustrate. The association most reliably detected is the amplification (log-ratio 2) spanning 20 probes, detectable in all situations. But as the total region size increases the proportion of affected probes decreases, with the effect becoming diluted, in particular if the sample size is small (25).

The effect least reliably detected is the one-copy gain due to the small amplitude (log-ratio 0.5): it is only detectable when 50% of 100 samples are affected and the region spans at least 500 probes. The one-copy loss is detectable in all situations with sample size at least 50. Note that, because the mild effects involve a fixed proportion of 26% of the probes, the power to detect them increases with the region length.

In practice, the amplitudes of recurrent copy number changes vary more, therefore associations between continuous copy number measurements and gene expression levels can also be detected for smaller sample sizes and less-frequent aberrations.

### Breast cancer I: Pollack

**Figure 2:**
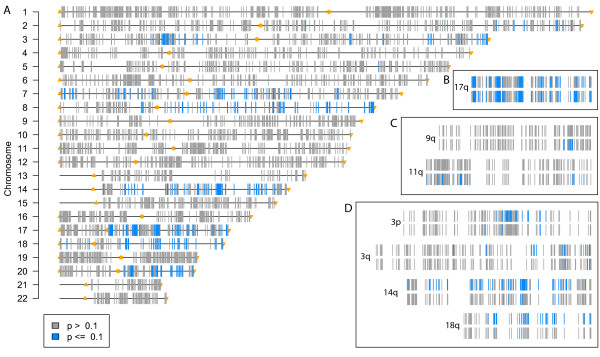
**Overview of associations found with Pollack's breast cancer data and the gene-set model**. Chromosomes are represented by horizontal bars, and the arms are the gene sets used, with gene set edges marked by triangles and stars. Each vertical bar represents one copy number probe. The colour of the bar indicates the test result: blue, significant(FDR ≤ 0.10); grey, not significant (FDR > 0.10). Results on the left-hand side (A) refer to all tests done on the Pollack data set using the gene-set model. Insets on the right-hand side display gene-set (top) and gene-to-gene (bottom) p-values for a selection of chromosome arms (B: 17q, C: 9q and 11q; D: 3p, 3q, 14q and 18q).

Pollack and co-authors [12] produced and were the first to analyse this dataset, consisting of copy number and expression array data for 37 breast tumors and 4 breast-tumor cell lines, produced on the same cDNA microarrays. The datasets pre-processed by the authors were downloaded, consisting of log-ratios per gene. The curated dataset involved 4696 genes with both copy number and expression log-ratios available. Here we wish to investigate if there are copy number changes that affect expression levels within the same chromosome arm. We report results controlling the FDR at 10%. Considering the copy number data on a continuous scale, we fit the gene-set model explaining measurements for each copy number probe by the expression levels of all probes on the same chromosome arm. This model found evidence of association between copy number and expression levels, with in total 343 probes significant out of 4696 (figure 2). The gene-to-gene model can be applied directly as the same microarray was used both for copy number and for expression. This model selected 272 clones, 114 of which also selected by the gene-set model (see figures 1 and 2A in Additional Files 1 and 2). This shows that the gene-to-gene model finds some of the same effects as the gene-set model, but each one finds unique effects: 158 by the gene-to-gene model, 229 by the gene-set model.

**Figure 3:**
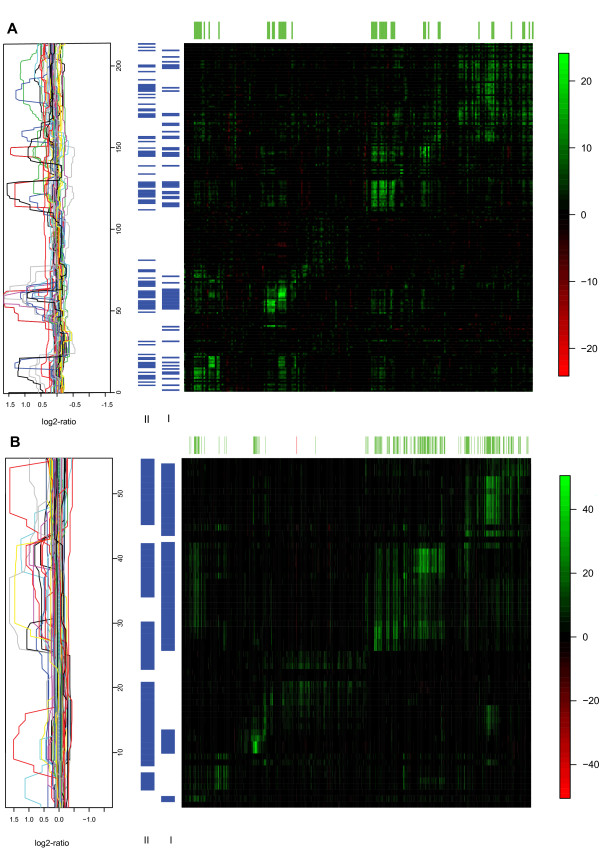
**Association patterns between copy number and gene expression found on 17q for two independent breast cancer studies**. Heatmap of association structure (green, positive; red, negative; black, no association) for the datasets of Pollack (A) and Chin (B), with rows representing copy number probes from centromere (bottom) to telomere (top), and columns representing gene expression probes from centromere (left) to telomere (right). The vertical bars on the left-hand side represent the p-values for the copy number probes, as calculated by the gene-set model (bar I) and by the window gene-to-gene model (bar II), with blue indicating the significant ones (for Pollack FDR ≤ 0.10, for Chin FDR ≤ 0.01). The top horizontal bar indicates expression probes with strong positive (green) or negative (red) association with the significant results from the gene-set model (mean z-score across significant tests ≥ 3). In the left panel, the log-ratio copy number values for all samples are represented by their smoothed medians, on the same probe spacing (equal space between each pair of consecutive probes) as used in the heatmap for comparability.

**Figure 4:**
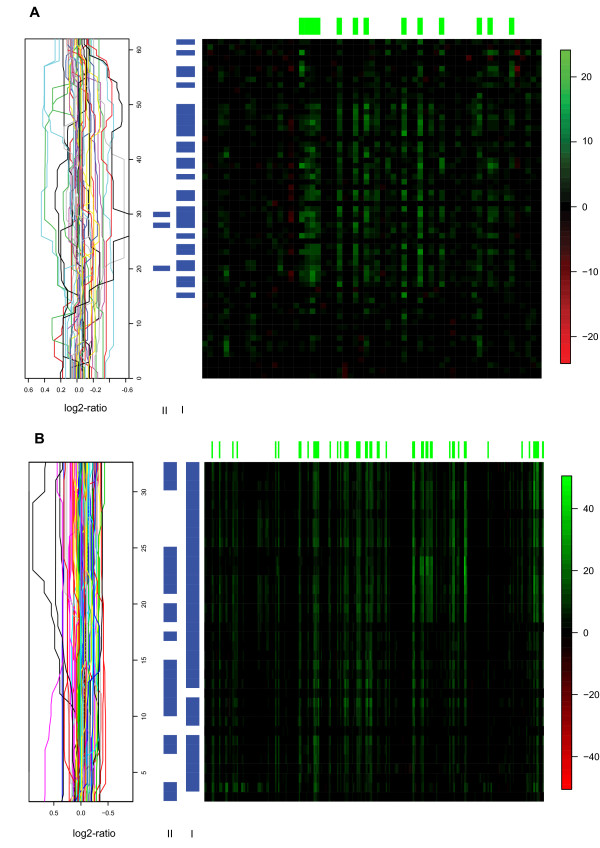
**Association patterns between copy number and gene expression found on 18q for two independent breast cancer studies**. Heatmap of associations for 18q. See figure 3 for a detailed description.

Some of the effects found by both models identify copy-number aberrant regions such as those on 8p, 8q, 17q and 20q (figure 2). In particular, 17q includes genomic regions with high-amplitude copy number effects (amplifications) highly associated with resident-genes' expression levels (figure 3A). One of these regions contains the *ERBB2 *gene, another one contains the *TRAF4 *gene. These genes were also found by Pollack [12], and are known to be involved in breast cancer development. Other candidate oncogenes were identified in gained regions 8p11-12 (including *LSM1*, *BAG4 *and *FGFR1*) and on 20q (including *NCOA3*). In other instances the models yield different results. In general, the gene-set model finds regions of association, i.e. it tends to find associations involving neighbouring copy number probes. In contrast, the gene-to-gene model focuses on effects on individual probes, so it often finds single probes with association with no other effects on neighbouring probes. On chromosome arms 3p, 3q, 14q and 18q (figure 2D), both models pick up at least one copy number probe as having association with expression within those arms, but the effects span genomic regions under the gene-set model, whereas they are restricted to individual probes under the gene-to-gene model. Looking in more detail at 18q, many samples have a mild copy loss of up to -0.4 on the median smooth scale, with a handful of samples displaying slightly larger losses (see figure 4A). A couple of samples display mild copy gain in the same region. It turns out that expression is affected, as indicated by the many statistically significant associations found by the gene-set model. The gene-to-gene model, however, only finds three of those associations statistically significant.

In general, the gene-set model particularly benefits from (mild) associations spanning multiple genes. If expression levels of resident genes are affected, many copy number probes mapping the aberrant region will show significant associations, thereby highlighting the region. On the other hand, copy number changes that affect only one or a few genes are picked up by the gene-to-gene model, but may become diluted when the entire chromosome arm is analysed by the gene-set model. For example, associations on 9q and 11q are only detected with the gene-to-gene model (Figure 2C).

### Breast cancer II: Chin

Let us now consider a set of 89 samples of breast tumor tissue, profiled both on a 2.5 K BAC array CGH and on an A3ymetrix U133A array from Chin and co-authors [29]. In this case there is no correspondence between copy number and expression probes, and the genomic coverage is rather different from the one yielded by Pollack's cDNA arrays, with the copy number arrays having about half the number of clones as in Pollack's data, and the expression arrays having over four times as many probes as Pollack's. Only probes with genomic annotation were used, totalling 2083 BACs and 21339 expression probe sets. Here we used again in the gene-set model all genes on a chromosome arm as a gene set. For all models applied to this dataset, associations found are those considered statistically significant with FDR control at 1%. This more strict threshold is used to make results more comparable with those from the previous example, where the number of samples (41) was less than half of the number of samples here.

First we fit overlap and window gene-to-gene models. The overlap gene-to-gene model, measuring association between copy number on BAC clones and expression probe sets included in it, yields 991 comparisons representing less than half of the copy number probes observed (2083). In contrast, the 2 Mb-window gene-to-gene model involves 2030 comparisons. So by considering only expression probes located within BACs more than half of the BACs is neglected. In addition, the associations considered by the overlap model are also considered by the window model by definition, unless the window used in the latter is smaller than the BACs, which is not the case here. So we expect to identify the same associations with both models, which indeed happens: of the 239 statistically significant associations identified by the overlap model, only 13 were not identified by the window model too. This is merely because, by involving a much smaller number of tests, the overlap model results involve a less severe multiple-testing correction. From this viewpoint the overlap model yields the same patterns as the window model, but the latter makes better use of the observed data. For this reason, we will focus hereafter on comparisons between the window gene-to-gene and the gene-set models. The genomewide associations found between copy number and gene expression with these two models can be seen in Additional File 3.

There are many associations found both by the gene-set and by the gene-to-gene model, but also associations found by only one (see figure 1B in Additional File 1). The many effects found by both models refer to those involving large enough copy number changes and/or expression changes. For example, we found a pattern of association on 17q with both models, very similar to what had also been found in the previous example (figure 3B). This may sound obvious, but it is less so considering the widely different microarray platforms used in the two studies, with markedly different genomic coverages, and the fact that independent samples are involved. Reflecting this, the number of probe sets mapping 17q differs markedly between the two studies: Pollack has 215 clones measuring both copy number and expression levels, whilst Chin has only 59 measuring copy number and as many as 913 measuring expression.

For some regions, the gene-set and gene-to-gene models yield different results. As in Pollack's data, for 18q the gene-set model finds clear association between copy number and expression for all but three clones in the region, in contrast with a weaker association detected by the gene-to-gene model (figure 4B). Indeed, copy number changes in this region are mild, with most changes being a loss of no more than 0.4 on the smoothed median scale. But many samples do display this loss, and its impact on expression drives the association. This mild effect is harder to be picked up by the gene-to-gene model.

An intermediate model between the gene-set on the chromosome arm and the gene-to-gene (on a 2 Mb window) models would be one that considers a smaller gene set, helping focus the search for associations in the region around the copy number probe. Such a gene set may be defined in various ways. Here we consider as gene set all gene expression measurements within a 2 Mb region centered around the copy number probe under study. As expected, this model finds many associations also identified by the other models, but also finds some more (see Additional File 4). Of the 2030 associations tested by all three models, 1344 (66%) were found to be statistically significant by at least one model and, of those, 519 (39%) were found by all three models. The largest overlap was found between the gene-to-gene and the gene-set on the 2 Mb window, with 775 associations found in common, which is reasonable. However, there were also associations found by each model individually, with the different models specializing in different effect types. This is further illustrated by re-examining significant associations found on chromosome 17 (Additional File 5).

### Population data: HapMap

Gene dosage, as copy number variation is often referred to in a biological context, plays a role in regulating gene expression in normal individuals, as was shown by the analysis of copy number and expression data from the HapMap2 samples [22]. Such effects tend to be milder than those found in cancer data, since copy number changes will be typically smaller in size here. Here we illustrate that the gene-set model also has the power to find associations in this context, where mild effects are measured by higher-density arrays. We have re-analysed the data using the gene-set model over each chromosome arm. For comparability with Stranger's results, our model explains the expression levels of each individual probe by the copy number values of all BAC clones in the same chromosome arm, in contrast with the first two examples. This means that around 18 K gene expression probes were tested for association with copy number. Our results yielded similar numbers of gene expression probes selected at the same p-value threshold, but the overlap with the probes selected by Stranger was relatively small (see supplementary table 1 in Additional File 6). A thoroughcomparison between our results and Stranger's is not our objectivehere. Rather we wish to show the added-value of the gene-set model compared to gene-to-gene models, such as the one used by Stranger. The gene-set model had more power to detect subtle associations that span at least a few probes. Indeed, we have identified a region on 6p where gene expression displays association with some of the copy number probes, within each of the populations. The region goes from 32.593 to 32.817 Mb and includes four expression probes. Using a gene-to-gene model, Stranger only picked up two of these four gene expression probes as being associated with copy number, and only for one of the four populations. The gain in power was thus significant by including many BACs in the model, in spite of the fact that the relevant association only involves a small number of BACs (four), compared to the total included in the model (607) on 6p. With the largest Pearson correlation between BAC clones and expression probes in this region being less than 80%, the effect seems not to be strong enough to be picked up using individual BACs as Stranger did. Note that the four expression probes selected map major histocompatibility complex class II genes, known to harbour polymorphisms that are commonly genotyped prior to organ transplants. Thus these known polymorphic areas have copy number-regulated gene expression, but that was only detected by the gene-set model.

## Discussion

We propose to jointly analyse DNA copy number and mRNA expression array data by modelling one (copy number, say) as a function of the values of the other (expression) for all genes in the same chromosomal arm or an independently defined region. This yields a gain in power to detect associations, as genome-based regulatory mechanisms tend to affect neighbouring genes. Considering the coordinate behaviour of groups of genes instead of individual genes was shown to be a useful strategy to improve robustness in gene expression analysis [30-32], but has not been previously used in the context of integrating expression data to another data type. Because the global test, the basis of our approach, has optimal power to detect subtle but consistent association between phenotype and expression signature [33], it enables us to detect associations between expression levels and low-level gains, in contrast with previous papers which had only been able to detect associations involving high-level gains [11,13,15,23,28].

Our approach is unique in several ways. Firstly, by considering the association between each probe in the dependent data and a gene set in the independent data, rather than a single gene, it stands a better chance of detecting subtle but consistent effects across many genes. Indeed, we have shown in a simulation study that subtle effects can indeed be found if at least 50 samples are studied. As key parameters of the study were estimated from tumor datasets, such as copy number and expression variability as well as their association, results can be extended directly to other studies. As further confirmation, associations between large- and small-amplitude copy number changes and gene expression levels were also found in the breast cancer datasets studied. The use of gene sets keeps focus on consistent changes that are unlikely to be data-dependent, as we showed by obtaining similar patterns of association for two independent datasets, in spite of widely different microarray platforms having been used.

Secondly, the use of a regression framework means that our model enables control of confounder effects. For the samples studied by Chin estrogen-receptor status was known. We applied the gene-set model using this variable as a confounder. Associations found were similar to those without considering the confounder for most chromosomes, except for five of them: for 1p, 5q, 6p and 12q, no features were selected with ER-status adjustment whilst the unadjusted model selected between 20% and 60% of the probes, and for 19q, no features were found with the unadjusted model, but about 40% of the BACs were selected with ER-status adjustment. More importantly, in each of these chromosome arms a handful of BACs was assigned an FDR-corrected p-value in one analysis below 0.01, whilst in the other the p-value was larger than 0.20. These results suggest that copy number-based mechanisms of gene expression regulation differ according to estrogen-receptor status in breast cancer.

Thirdly, our model can be used with continuous copy number, as log-ratios like in our examples or on the original copy number scale, as well as segmented or discretized copy number data. Here we point out that there is no consensus as to whether or not association testing would benefit from segmentation of DNA copy number data [22,24,34]. However, we recommend using continuous data at least in cancer studies, because a non-integer number of copies may represent the average number of copies found on the sample of cells collected for that particular tumor, in which case a sharp cut-off is likely to introduce a bias.

Finally, our approach avoids introducing bias via matching between copy number and expression probes on the genome, as it rightly focuses on finding relevant associations regardless of their genomic location. We compared the performance of the gene-set model to that of the gene-to-gene model. While the former has more power to identify regions with coordinated association, the latter yields individual associations unrelated to possible effects in its neighbourhood, as expected. Associations involving a large proportion of samples displaying a large-amplitude copy number change are typically picked up by both models, as is the case on 17q, known to harbour regions of large amplifications that play a causal role in breast cancer. Mild associations, because either the copy number change has small amplitude, or the effect on gene expression is limited, or even the proportion of samples involved is small, are less likely to be identified by the gene-to-gene model than by the gene-set model. A clear example is that of 18q, where in both examples the gene-set model identifies the effect spanning a large region, but the gene-to-gene model just selects a handful of probes. Note, however, that the overlap between results of the two models for the Chin dataset is positively associated with window size and, as the 2 Mb window size used included most of the associations available, considerable overlap was the result.

From the biological viewpoint, associations found for many copy number probes within the same region are reassuring, as they are less likely to be driven by pure noise. Because it tends to find regions of association, the gene-set model is more robust to noise than the gene-to-gene model.

These two models represent two extremes. An intermediate model may be used to diminish the dilution, whilst still being robust to noise. Such a model could be a gene-set model over a window centered around the copy number probe, as used in the Breast Cancer II example. This sort of model is particularly useful when interest lies in mild associations, either spanning small regions, involving low-amplitude copy number changes or having a limited impact on expression levels. It might be particularly useful when high-density arrays are involved. Nevertheless, the gene-set model remains the least arbitrary and, while dilution might be a concern, individual effects may still be identified by visual inspection of heatmaps representing association patterns found. In such cases, focus shifts from finding statistically significant associations to finding consistent association patterns between copy number and expression. The nature of the problem turns then from that of hypothesis testing to an exploratory one.

We considered two ways in which the gene-to-gene model (1) can be used in a study where different microarray platforms were used to measure copy number and expression. The first one involved considering only copy number and expression probes that overlap, so that only measurements for the same locus are considered. The second way was to calculate associations between each copy number probe and expression probes located within a certain distance from it. Another possible way is to interpolate the copy number measurements, say using quantile smoothing as suggested by Eilers and Menezes [35], and thus obtain copy number estimates corresponding to all loci for which expression was measured. This would avoid the problem from the first approach that non-overlapping probes are neglected, and the arbitrariness of defining a distance on the second approach, so making use of all observed measures. However, it relies on good approximations via interpolation. If the density of the copy number probes is high with reasonably small intervals between probes, interpolated values tend to estimate well the true copy number. On the other hand, with large between-probe distances such as 1 Mb, this is less likely to be the case. In all cases some arbitrariness is involved, which the gene-set model avoids.

The gene-set model can be formulated in alternative ways to answer different questions. Perhaps the most intuitive formulation is to use expression as outcome and copy number as explanatory variable, best suited to find genes which expression is regulated by copy number changes in the region around it. However, if the objective is to find DNA-based markers that regulate gene expression on the same region, then the best formulation uses copy number as outcome and expression as explanatory variable, as we did in the analysis shown here. By considering the expression values of many genes simultaneously, this formulation is also able to capture coordinated variability in expression levels across genes, such as co-regulation, which would not be possible otherwise due to noise. This is relatively less important in copy number data, which typically displays relatively less noise compared to the signal.

It is straightforward to extend the model to analyse other types of high-dimensional data. For example, another type of expression regulation mechanism is DNA methylation, which can be measured via CpG-island arrays. In a similar way to simultaneous analysis of copy number and expression array data, there could be interest in analysing DNA-methylation and expression. The use of gene sets are still likely to improve power to detect associations, as DNA-methylation may affect the expression levels of multiple genes, like copy number.

A second interesting extension is to consider more than two types of array data in model (2). For example, gene expression can be regulated by different mechanisms in addition to copy number, including transcription factor levels, sequence changes, DNA methylation, loss of heterozygosity, and chromatin structure. Our method can be generalized to analyze the association between gene expression and other types of genomic information simultaneously. This extension is beyond the scope of this paper and will appear elsewhere. We hope that such a model taking into account multiple data sources simultaneously will shed light on the influence of different genetic and epigenetic mechanisms on gene regulation.

Finally, while the gene-set model serves as a starting point to identify copy number changes that are associated with expression patterns, additional experiments are needed to validate the possible role of these changes in the causation or maintenance of the phenotype under study.

## Conclusion

We have proposed, and given proof of principle for, a new approach to identify association between high-throughput genomic copy number and gene expression profiling data, which can be used to identify putative candidate genes involved in tumorigenesis. By considering the expression levels of many genes simultaneously in the model, our approach identifies regions of association even if low-amplitude copy number changes are involved. The regression is able to control for confounder effects. Finally, it requires neither matching between copy number and expression probes on the genome, nor categorization of copy number, both of which are possible sources of bias.

## Methods

We assume that each sample is profiled both on a copy number and on an expression array, that the copy number and the expression array data were separately pre-processed, adequately normalized and that probe annotation including identifier, chromosome number and location in base pairs is available.

We shall focus on answering the following question: which copy number changes affect gene expression within the same chromosomal region? This question typically arises when searching for DNA-based markers that regulate expression via copy number change.

### The gene-to-gene model

Since our main interest is to find DNA-based markers that are associated with expression changes, it makes sense to consider copy number as the dependent variable, so expression is handled as the independent variable. The simplest model to consider is

(1)

where *Y*_*ni *_represents the copy number measured for sample *n *and array-CGH copy number probe *i *(*i *= 1,...,*I*) and *X*_*ni *_represents the expression level for sample *n *and expression probe *i*. This model is written assuming that there is a one-to-one correspondence between copy number and expression probes, for simplicity, which holds for example if the same array is used to measure both copy number and expression levels. Such a model is especially useful if, in addition to using the same array, the study goal is to find associations between copy number change and expression variation at the same locus. We shall refer to (1) as the *gene-to-gene *model.

In many cases interest lies in studying effects of copy number change on expression of resident genes, i.e., genes within the same region where copy number was measured. But it can be the case that not the same array was used to measure copy number and expression. Then model (1) can still be used if the data and/or the model are adapted. The first thing that can be done is to consider only expression probes that fall within copy number probes, so as to ensure that measurements relate to the same locus. We refer to this as the *overlap *approach. This is rather strict, and may result in many expression probes not being considered, even if they are close to the copy number probe. To relax this, we can consider expression probes that are within a certain distance from the middle of the copy number probe in each direction, and then fit model (1) to each one separately. We refer to this as the *window *approach, where the window size is the length of the entire interval considered. This may yield more than one test for some copy number probes, whilst for others with no expression probes near it no tests are done. So some arbitrariness is involved in defining the distance, which directly affects which tests are considered. Here we shall use a window of size 2 Mb (similar to Stranger *et al*. [22]), centered around the start of the copy number probe.

### The gene-set model

In practice, it is not commonly the case that the same array platform is used for copy number and expression. Moreover, the arbitrariness of the window definition is undesirable, and considering only overlapping probes leads potentially to loss of valuable information. An ideal way to avoid these problems is to include in the model all expression probes within the same large region. This leads us to the model, for each copy number probe *i*,

(2)

By considering the expression levels of many genes simultaneously, this model suits well most situations where copy number changes produce an effect spanning many expression probes, in a possibly subtle but consistent way. Because (2) makes use of a set of genes as independent variables, we shall refer to it as the *gene-set *model.

Note that the gene-set model (2) is not estimable if *J *> *n*, in a classic linear regression context. Since our main objective is to test whether copy number change is associated in general with expression levels {*X*_*nj*_, *j *= 1,...,*J*}, it is natural to study the distribution of *β *≡ (*β*_1_,...,*β*_*J*_)^*t*^, a vector of independent random variables. We assume that each *β*_*j *_has a certain distribution and, under the null hypothesis of no association between *X *and *Y*, has mean 0 and variance *τ*^2 ^≡ 0. The assumption *β *~ (0, *τ*^2^*I*_*J*_) means that model (2) is a random-effects model, and a natural distribution to assign to the vector *β *is the multivariate normal with a covariance matrix *τ*^2^*I*_*J*_, where *I*_*J *_represents the identity matrix with *J *rows. The random-effects model framework arises thus naturally from the question under study and the biological context. Moreover, it guarantees that model (2) is identifiable, which would not be the case in the classic linear regression model framework if *J *>> *N*, which is often the case. Thus, model (2) can be fitted using methods for random-effects models.

Under the alternative hypothesis of association, the mean of each *β*_*j *_may still be zero, but their variance should be strictly positive (*τ*^2 ^> 0), suggesting that a non-empty subset of the {*X*_*nj*_, *j *= 1,..., *J*} is associated with copy number measurements for probe *i*. Therefore, we shall focus on testing *H*_0 _: *τ*^2 ^= 0 against *H*_*a *_: *τ*^2 ^> 0. A test to compare such null and alternative hypotheses was proposed by [30] for testing association between expression levels of a set of genes, e.g. those belonging to a biological pathway, with a clinical outcome. This approach has been shown to have more power to detect subtle associations than by performing separate tests and correcting the resulting p-values for multiple testing [33]. We shall make use of this global test as the basis for our approach in this new context, as well as consider extensions of interest.

By modelling the copy number at each locus by expressions within a large gene set, the gene-set model (2) takes advantage of the typically larger signal-to-noise ratio in copy number compared with gene expression microarray data. By considering the expression levels of many genes jointly, coordinated expression changes, such as co-regulation, are more likely to be detected than if gene expression levels were considered separately.

### Considering covariates

Because models (2,1) are constructed within a regression framework, other explanatory variables which can act as confounders can be included. This is very important in more complex designs, such as when more than one sample is collected per patient, when patients are related, or when clinical variables are to be taken into account, for example tumor location and age.

Note that, as in multivariate regression analysis, the inclusion of a confounder can weaken or even eliminate an effect, if the association is limited to one confounder-defined subgroup. On the other hand, it may explain part of the copy number variation bringing out new associations.

### Multiple testing correction

Both models (1) and (2) yield one *p*-value per copy-number probe tested. Copy number levels of neighbouring probes are likely to be associated, but genomic breaks such as centromeres and telomeres may break this association. Therefore, it seems reasonable to treat chromosomal arms independently in the analysis, including in what concerns multiple testing correction. The correction must allow for dependency between the tests, and currently the most adequate method available has been suggested by [36].

Note that the overlap and window approaches used with the gene-to-gene model (1) involve different numbers of tests, implying different multiple testing corrections, unless of course the same microarray is used to measure both copy number and expression. Indeed, the overlap approach typically will involve a smaller number of tests than there are copy number probes, in contrast with the window approach which may generates a larger number of tests than there are copy number probes.

### Definition of genomic region

The length of the genomic region under study may affect the results of the model fit via the amount of multiple-testing needed and, to a lesser extent, via the number of explanatory variables, i.e. gene expressions, in the model. To avoid introducing bias in this way, we suggest that the genomic regions to be studied be determined *a priori*. In our experience chromosome arms are sensible such regions, as are minimal common regions of recurrent copy number aberrations.

### Visualization and prioritization of genes

For each copy number probe, the test statistic can be decomposed into the individual contributions of the genes' expression levels [30]. After standardization per probe, we display the separate contributions of the gene expressions to copy number variability by means of a heatmap, where rows and columns represent copy number and expression probes respectively, both kept in their genomic order. If a copy number change spanning roughly the same genomic region across a subset of samples is positively associated with gene expression on the same region, it will be represented by a green rectangle on the diagonal. For each copy number probe, discretised *p*-values computed with the test can be displayed as a vertical bar next to the heatmap, so that significantly associated genomic regions are highlighted.

It is often of interest to identify the gene expression probes with the largest contributions to the test results, in some sense. We choose to compute the mean standardized contribution over all significant tests, per expression probe, then rank them to generate candidate genes for future investigation. This yields candidate genes whose expression levels are highly associated with copy number. By considering only the significant tests, we want to avoid diluting a possible association spanning a relatively small area, compared to the entire area under study.

### Software used

We have used R version 2.5.1 [37] for all our analyses. In addition, we used the following R packages: globaltest, marray, multtest and quantsmooth. An R package called SIM implementing this approach has been made available via BioConductor.

## Authors' contributions

RXM developed the concept, was involved in the statistical analyses, and wrote the manuscript. JMB helped develop the concept, was involved in interpretation of the results, and revised the manuscript. MS and MB performed part of the analyses and participated in developing the BioConductor package SIM. GJVO participated in the study design and coordination. All authors read and approved the final manuscript.

## Appendix

### Simulation study setup

#### Assumptions

In order to evaluate how the regional integration model works, we run a simulation study. For this, we assume for simplicity that both copy number and expression measurements are obtained using the same arrays, so that there is a one-to-one correspondence between them. We also assume that the copy number is measured in terms of log-ratios, that the expression is measured in terms of intensities, and that the datasets were normalized separately as adequate. All these assumptions are made for the sake of simplicity, being unimportant for the qualitative results of this study.

For each probe *i *= 1,...,*I*, the data consists of copy number measurements *Y*_*i*1_,...,*Y*_*iN *_and expression measurements *X*_*i*1_,...,*X*_*iN *_for samples 1,...,*N*. We assume that samples are independent and that data distribution is independent of sample, so we ignore the sample index from now on for simplicity. The true log-ratio copy number of gene *i *is represented by *ξ*_*i*_. We represent by *Z*_*i *_the binary indicator variable that copy number of gene *i *regulates its expression, so that the vector *Z *= (*Z*_1_,...,*Z*_*I*_)^*t *^represents expression-regulating copy number regions. We assume that there are three such regions: region I, spanning 20 probes which all have true copy number 5, so a gain of 3 copies; region II, spanning around 26% of the probes which all have true copy number 3, so a gain of one copy; and region III, spanning also 26% of the probes which all have true copy number 1, so a loss of one copy.

Copy number measurements *Y*_1_,...,*Y*_*I *_are assumed to be independent, each *Y*_*i *_with distribution **N**(*ξ*_*i*_, ). Note that if gene *i *is located in any of the three regions of expression-regulating copy number, *Z*_*i *_= 1 and *ξ*_*i *_≠ 0 and, for all genes outside these regions, *Z*_*i *_= *ξ*_*i *_= 0.

Expression measurements *X*_1_,...,*X*_*I *_are assumed to be conditionally independent, given *Y*_1_,...,*Y*_*I*_, with (*X*_*i*_|*Y*_*i*_)~**N**(*μ*_*i*_{1+*α*_*i*_*ξ*_*i*_}, ), where *μ*_*i *_represents gene *i*'s baseline expression level, and *α*_*i *_is a variable representing the extent to which copy number of gene *i *regulates its expression.

### Parameter values

The mean log-ratios of copy numbers *ξ*_*i*_|*Z*_*i *_= 1 were estimated from [38], which made use of commercially available DNA samples with known copy number. These data yielded *ξ*_*i *_= 2, *ξ*_*i *_= 0.5 and *ξ*_*i *_= -0.85 for genes in regions I, II and III respectively. Note that there is signal compression of the expected amplitude. Log-ratios of copy numbers {*Y*_*i*_, *i *= 1,...,*I*} were drawn independently from a *N*(*ξ*_*i*_, ) where its dispersion 1/ follows a Γ(2, 2), so the mean dispersion is 4. Given *Y*_*i*_, *μ*_*i *_is drawn from a *N*(9.5, 2.3^2^), independently for each *i*. The dispersion of *X*_*i*_|*Y*_*i*_, 1/, is in its turn drawn from a Γ(2, 0.5), implying that the mean dispersion is 1.

Copy-number impact on expression per probe *i*, *α*_*i*_, was estimated using three subregions of the breast cancer data from [12], as in this case the same array was used to produce both copy number and expression. These three regions were chosen so as to contain copy number changes, some of them having been found to be associated with expressions, and were located in chromosomes 1q, 8p and 17q. First of all, the relationship between copy number and expression for the 303 selected probes can be described reasonably well by a linear function (data not shown). Moreover, the normal distribution seems to yield a reasonable approximation to the empirical distribution of *α*_*i*_, with mean and variance estimated as -4.6 and 8.3, respectively (data not shown). So, in our simulation we draw *α*_*i *_from this distribution. Other parameters that need to be fixed and values used are given in supplementary table 2 (see Additional File 6).

Note that the direction of the effect (positive or negative) is unimportant for its detectability by the model, only the log-ratio is important.

## Supplementary Material

Additional file 1 **Overview of associations found with Pollack's breast cancer data and the gene-to-gene model**. Results obtained with the gene-to-gene model, where association is measured between each pair of copy number and gene expression measures obtained with the same cDNA clone. Chromosomes are represented by horizontal bars, with telomeres and centromeres marked by triangles and stars respectively. Each vertical bar represents one copy number probe. The colour of the bar indicates the test result: blue, significant(FDR ≤ 0.10); grey, not significant (FDR > 0.10). Click here for file

Additional file 2 **Venn diagram of associations found by two models in two independent breast cancer studies**. Overlap of associations between copy number and expression found significant by the gene-set (right, in blue) and gene-to-gene (left, in red) models. For the gene-set model, the chromosome arm was used as gene set. A – Pollack's data (FDR ≤ 0.10); B – Chin's data (FDR ≤ 0.01), for which the gene-to-gene model was applied on a 2 Mb window. Click here for file

Additional file 3 **Overview of associations found with Chin's breast cancer data**. Chromosomes are represented by horizontal bars, with centromerers and telomeres marked by triangles and stars. Each vertical bar represents one copy number probe. The colour of the bar indicates the test result: blue, significant (FDR ≤ 0.01); grey, not significant (FDR > 0.01). A: gene-set model; B: gene-to-gene model. Click here for file

Additional file 4 **Venn diagram of associations found by three models with Chin's breast cancer data**. Overlap of associations between copy number and expression found significant by the gene-set model using chromosome arm (right), the gene-set model using only gene expression probes on a 2 Mb window around the copy number probe (bottom) and the gene-to-gene model applied to the same 2 Mb window. Significance threshold was taken as FDR ≤ 0.01. Click here for file

Additional file 5 **Detail of associations found on 17q by three models with Chin's data**. Each vertical bar represents one copy number probe, and each horizontal bar one model: gene-set model using chromosome arm (top), gene-set model using only gene expression probes on a 2 Mb window around the copy number probe (middle) and gene-to-gene model applied to the same 2 Mb window (bottom). A: region where associations are found only with the models considering the 2 Mb window; B: region where associations are found only with the model considering the entire chromosome arm as gene set. Click here for file

Additional file 6 **Supplementary tables: 1. Number of gene expression probes associated with copy number in HapMap data; 2. Parameter values used in the simulation study**. In table 1, the significance threshold used for uncorrected p-values was 0.001, for comparability with Stranger et al (2007). Click here for file
