# Supplementary figures and images for: Integrated analysis of DNA copy number and gene expression microarray data using gene sets
Source: BMC Bioinformatics. 2009 Jun 29;10:203. doi: 10.1186/1471-2105-10-203 (PMC2753845; doi:10.1186/1471-2105-10-203)

## Significantly associated features

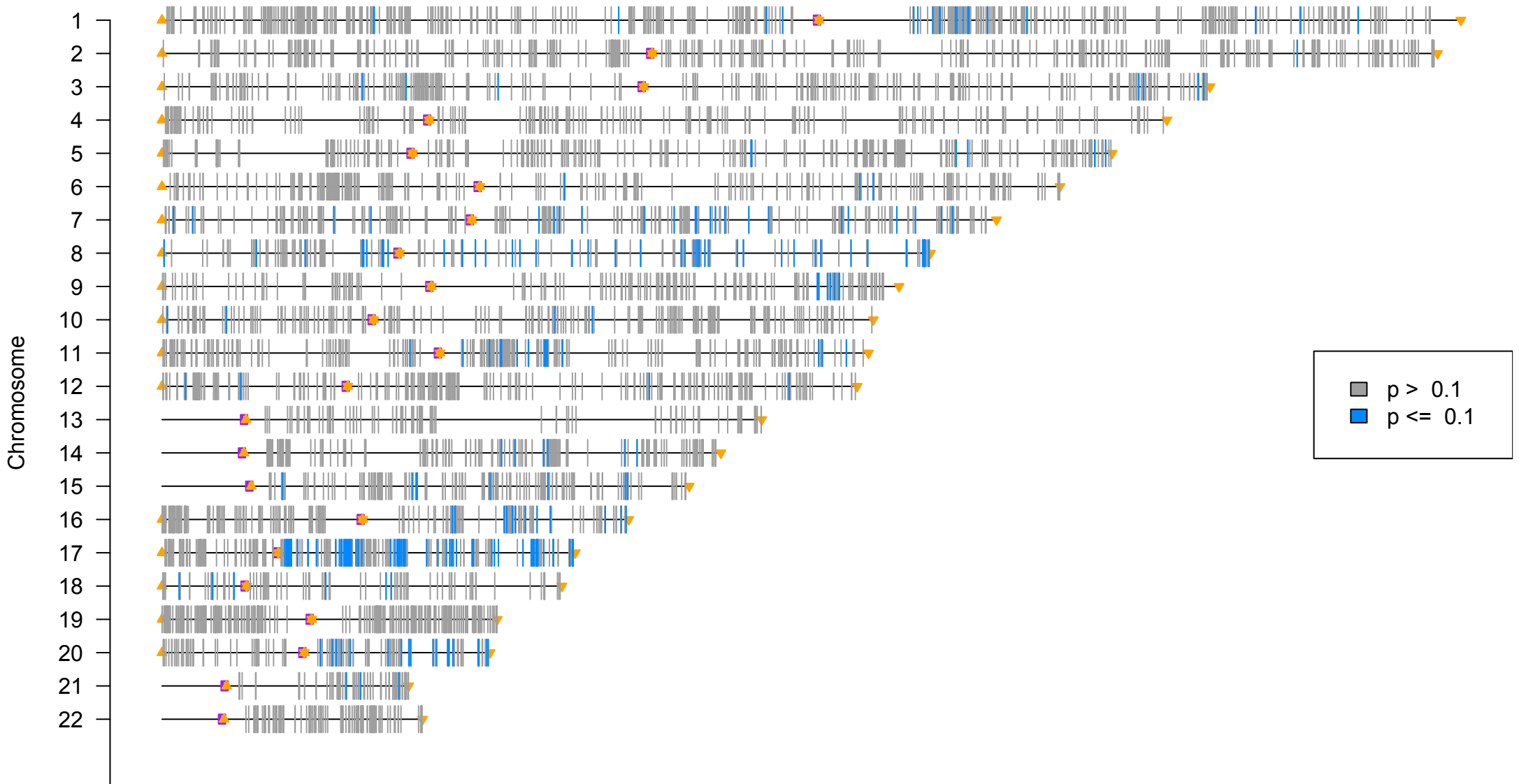

Supplement: Additional file 1 — Overview of associations found with Pollack's breast cancer data and the gene-to-gene model. Results obtained with the gene-to-gene model, where association is measured between each pair of copy number and gene expression measures obtained with the same cDNA clone. Chromosomes are represented by horizontal bars, with telomeres and centromeres marked by triangles and stars respectively. Each vertical bar represents one copy number probe. The colour of the bar indicates the test result: blue, significant(FDR ≤ 0.10); grey, not significant (FDR > 0.10). [file 1471-2105-10-203-S1.pdf]

gene-to-gene

gene-set

A

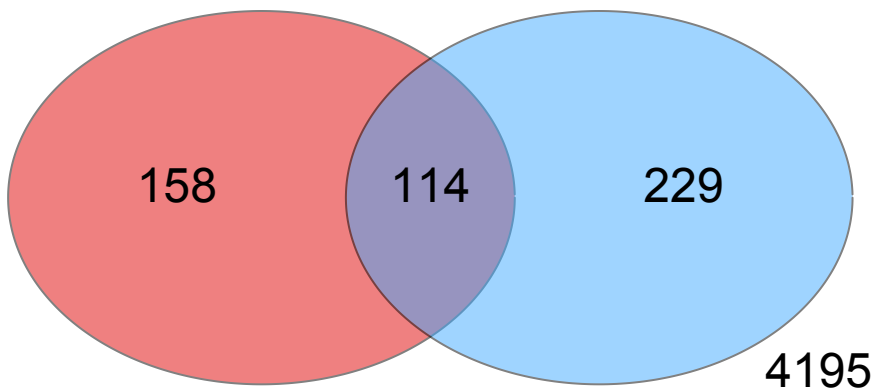

B

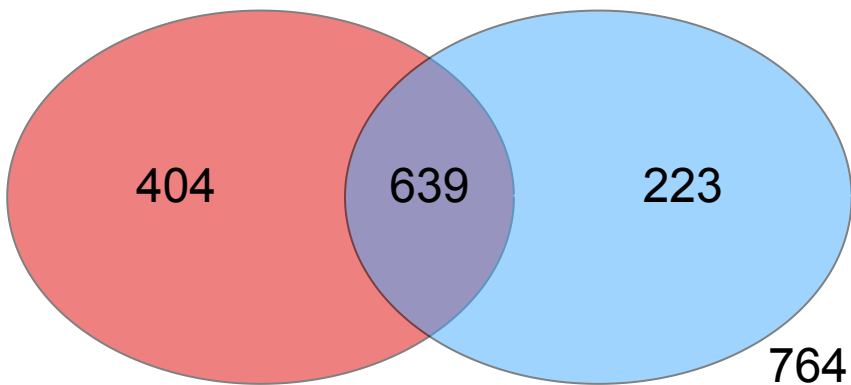

Supplement: Additional file 2 — Venn diagram of associations found by two models in two independent breast cancer studies. Overlap of associations between copy number and expression found significant by the gene-set (right, in blue) and gene-to-gene (left, in red) models. For the gene-set model, the chromosome arm was used as gene set. A – Pollack's data (FDR ≤ 0.10); B – Chin's data (FDR ≤ 0.01), for which the gene-to-gene model was applied on a 2 Mb window. [file 1471-2105-10-203-S2.pdf]

A

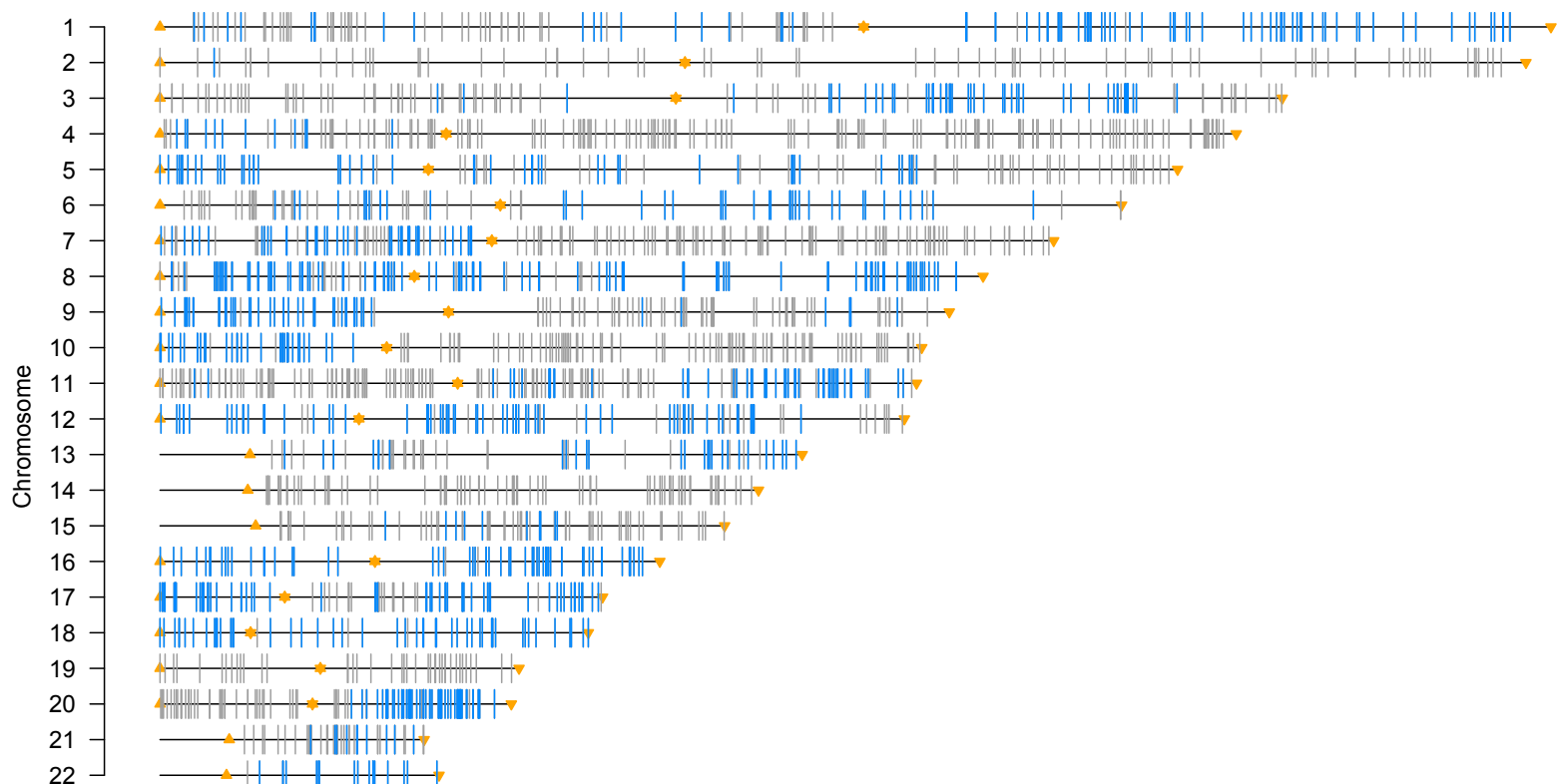

B

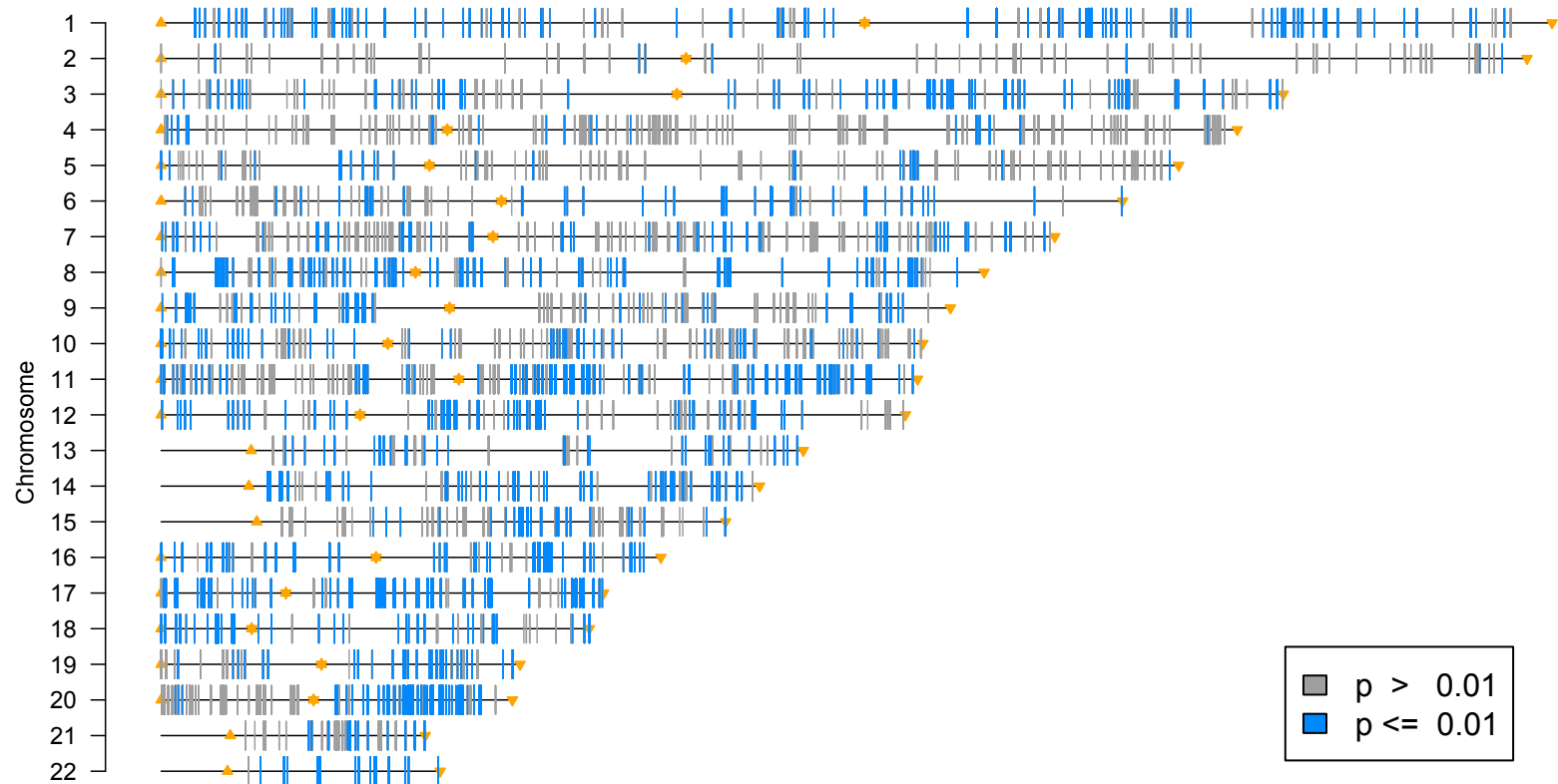

Supplement: Additional file 3 — Overview of associations found with Chin's breast cancer data. Chromosomes are represented by horizontal bars, with centromerers and telomeres marked by triangles and stars. Each vertical bar represents one copy number probe. The colour of the bar indicates the test result: blue, significant (FDR ≤ 0.01); grey, not significant (FDR > 0.01). A: gene-set model; B: gene-to-gene model. [file 1471-2105-10-203-S3.pdf]

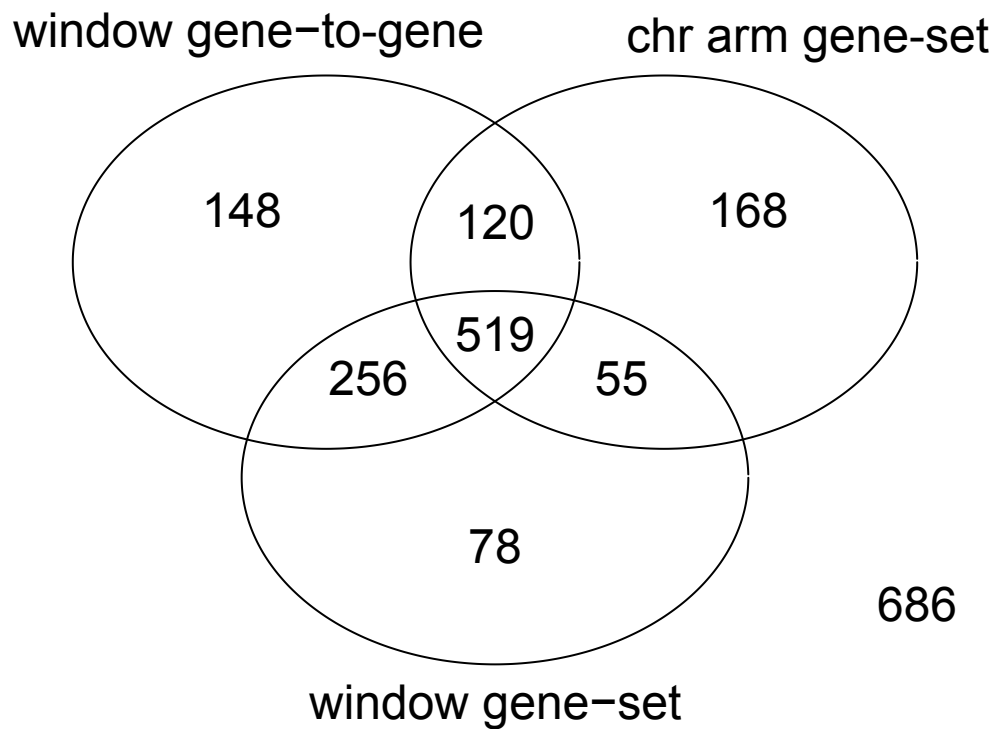

Supplement: Additional file 4 — Venn diagram of associations found by three models with Chin's breast cancer data. Overlap of associations between copy number and expression found significant by the gene-set model using chromosome arm (right), the gene-set model using only gene expression probes on a 2 Mb window around the copy number probe (bottom) and the gene-to-gene model applied to the same 2 Mb window. Significance threshold was taken as FDR ≤ 0.01. [file 1471-2105-10-203-S4.pdf]

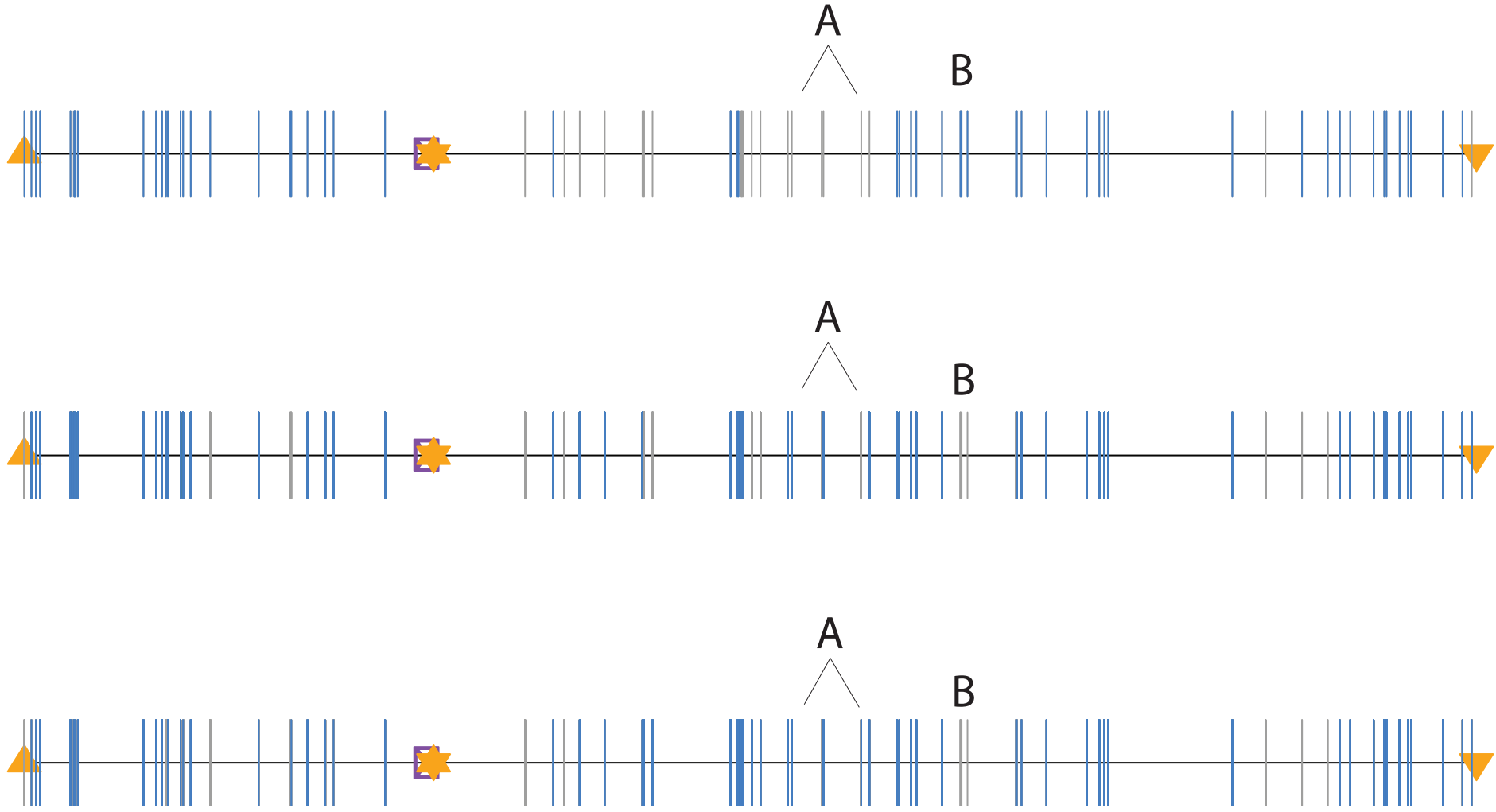

Supplement: Additional file 5 — Detail of associations found on 17q by three models with Chin's data. Each vertical bar represents one copy number probe, and each horizontal bar one model: gene-set model using chromosome arm (top), gene-set model using only gene expression probes on a 2 Mb window around the copy number probe (middle) and gene-to-gene model applied to the same 2 Mb window (bottom). A: region where associations are found only with the models considering the 2 Mb window; B: region where associations are found only with the model considering the entire chromosome arm as gene set. [file 1471-2105-10-203-S5.pdf]
