# Supplementary material for: Integrated analysis of DNA copy number and gene expression microarray data using gene sets
Source: BMC Bioinformatics. 2009 Jun 29;10:203. doi: 10.1186/1471-2105-10-203 (PMC2753845; doi:10.1186/1471-2105-10-203)
Supplement: Additional file 6 — Supplementary tables: 1. Number of gene expression probes associated with copy number in HapMap data; 2. Parameter values used in the simulation study. In table 1, the significance threshold used for uncorrected p-values was 0.001, for comparability with Stranger et al (2007). [file 1471-2105-10-203-S6.pdf]

Supplementary Tables

Supplementary Table 1 - Number of gene expression probes associated with copy number in HapMap data. Significance threshold for uncorrected p-values was used as 0.001, for comparability with Stranger et al (2007).

Population	Gene-set model	Stranger	Overlap
CEU	61	85	8
CHB	48	44	6
JPT	96	58	6
YRI	112	96	8

Supplementary Table 2 - Parameter values used in simulation

Definition	Notation	Values		
Region length	I	250	500	1000
Sample size	N	25	50	100
Proportion affected samples	π	0.10	0.25	0.50
